# The Plant Steroid Hormone, Epibrassinolide, Reverses Drug Resistance in Small-Cell Lung Carcinoma Cells by Modulating Ferroptosis

**DOI:** 10.3390/cancers18162659

**Published:** 2026-08-18

**Authors:** David Sadava, Shiuan Chen

**Affiliations:** Department of Cancer Biology and Molecular Medicine, Beckman Research Institute, City of Hope, 1500 East Duarte Rd., Duarte, CA 91010, USA; schen@coh.org

**Keywords:** small-cell, lung cancer, ferroptosis, drug resistance, epibrassinolide, Akt signaling

## Abstract

**Background:** Small-cell lung cancer (SCLC) has a poor prognosis because of the development of drug resistance. Our previous studies showed that the plant steroid hormone, epibrassinolide (EB), interacts with human SCLC cells through inhibition of GSK3β-mediated signaling and overcomes drug resistance. However, the latter does not occur via membrane-mediated drug efflux, a commonly reported mechanism. **Methods:** We investigated a novel mechanism, ferroptosis, for drug resistance and the effects of EB. Pharmacological, cellular and molecular investigations were performed. We used drug-sensitive (NCI-H69) and multi-drug-resistant (VPA) SCLC cell lines. **Results:** 4-hydroxynonenal (HNE), a marker for lipid peroxidation and ferroptosis, was lower in drug-resistant VPA cells than drug-sensitive cells (H69) (22 vs. 60 pg/mg protein). Incubation with EB at a dose that reverses drug resistance in VPA cells increased HNE (94 pg/mg). Exogenously applied HNE increased intracellular HNE and reversed drug resistance (IC_50_ for etoposide in VPA cells at 7.5 µM (untreated) vs. 1.0 µM (treated)). Ferrostatin, which inhibits ferroptosis, reduced HNE accumulation and increased drug resistance (IC_50_ for etoposide at 5.5 µM (untreated) vs. 19 µM (treated)). As measured by phosphorylation, the signaling molecule Akt was more active in drug-resistant compared to drug-sensitive SCLC cells. This activation was accompanied by increased drug resistance. Both EB and a specific inhibitor of Akt phosphorylation/activation reversed this resistance. Akt activation was reflected in increased GSK3β activity. The latter was also inhibited by incubation in EB. The level of the GSK3β-controlled “antioxidant transcription factor” NRF2 was higher in drug-resistant VPA cells than drug-sensitive H69 cells. This increase was reversed in EB-treated cells. The protein level of the “antioxidant enzyme”, GPX4, which chemically reduces peroxidized lipids, was higher in VPA than H69 cells (21.2 vs. 10.1 pg/mg protein). Likewise, GPX4 enzyme activity was higher in drug-resistant than drug-sensitive cells. Incubation with EB reversed these increases. Incubation of SCLC cells with RSL3, a specific inhibitor of GPX4 activity, increased intracellular HNE and reversed drug resistance in VPA cells. **Conclusions:** These data indicate that ferroptosis is an important mechanism for drug resistance in SCLC cells and the plant steroid, EB, reverses drug resistance by interfering with the Akt signaling pathway and GPX4 anti-oxidant activity.

## Simple Summary

Small-cell lung cancer (SCLC) accounts for about one in eight lung cancers and is especially lethal, with few patients surviving 5 years after initial diagnosis. Because the cancer has usually spread at diagnosis, treatment typically involves combinations of chemotherapy drugs. While these drugs work at the start, tumor resistance usually develops. There is a need to develop new strategies to overcome this resistance. We showed that in human SCLC cells, there is a novel mechanism called ferroptosis that accounts for resistance. Ferroptosis involves the accumulation of oxidized fatty acids in membranes that are harmful to the cells, and our studies demonstrated that in resistant SCLC cells, these toxic chemicals do not accumulate. We showed that a widely consumed, non-toxic plant hormone, epibrassinolide (EB), can overcome resistance by permitting ferroptosis so that the cancer cells will die.

## 1. Introduction

Lung cancer is the leading cause of cancer diagnoses and deaths worldwide. According to GLOBOCAN, there are approximately 2.6 million lung cancer diagnoses (12.8% global annual incidence) and 1.9 million deaths (19.1% global deaths). Small-cell lung cancer (SCLC) is a neuroendocrine tumor that accounts for approximately 12% of all lung cancer, with an annual incidence in the US of 4.5 cases per 100,000 [1]. SCLC is generally associated with tobacco smoking (95% of patients), and with reductions in this activity, the incidence of SCLC is slowly declining [2]. Unfortunately, this tumor is aggressive, with rapid cell proliferation and often with disseminated disease at initial presentation. Typically, SCLC has a 5-year survival of less than 15%. Although many genetic abnormalities have been identified in SCLC, including mutations in *TP53* and *RB1*, it has not yet been as responsive to molecularly targeted therapies as its more common organ counterpart, non-small-cell lung cancer (NSCLC) [3]. Therapeutic approaches targeting topoisomerases, HDAC, PARP and Chk1 are being introduced with limited success, and clinical trials are ongoing [4].

A major challenge in treatment of SCLC is the development of multi-drug resistance [5]. In general, cancer drug resistance has been considered to result from several mechanisms, including over-expression of drug efflux pumps such as ABCB1 (*pgp*) and ABCC1 (*mrp1*)*,* both of which are expressed in drug-resistant SCLC; enhanced DNA repair; and expression of anti-apoptosis genes such as *BCL2* [6]. More recently, research has focused on the roles of Akt signaling [7], ferroptosis, and anti-oxidation pathways in drug resistance in other tumor types, but heretofore not SCLC [8,9,10,11]. SCLC cells express the transferrin receptor and accumulate iron, a prerequisite for ferroptosis [12].

Brassinosteroids are a class of hormones present in many tissues of all taxonomically higher plants, where they have roles in plant development and responses to abiotic stresses [13,14]. While the molecular pathways for activities of these hormones have been described in plants, their widespread distribution in plants that are consumed in the human diet, as well as similarities in plant and animal signaling pathways with the latter important in cancer, prompted investigations into the effects of the most common of these hormones, epibrassinolide (EB), on human cells, particularly SCLC cells. Our previous studies showed that when applied to SCLC cells, EB is cytotoxic and pro-apoptotic, reduces the expression of SCLC tumor markers and inhibits cell signaling and proliferation via glycogen synthase kinase 3 (GSK3β) [15,16]. Of particular interest is that EB reverses drug resistance. However, it does not appear to do so via drug efflux pumps, as both the expression of genes encoding these pumps and protein levels are unchanged in EB-treated cells that are resistant. These results prompted investigations of a different mechanism possibly involved in drug resistance, ferroptosis. Our previous studies showed that SCLC cells express the transferrin receptor, a prerequisite for the accumulation of Fe^2+^ in ferroptosis.

Analysis of tumor samples indicates that the Akt signaling pathway is activated in drug-resistant SCLC [7]. The mechanism by which this pathway and drug resistance are connected has been investigated in several tumor types, but not SCLC [17]. Briefly, Akt signaling acts through the inactivation of GSK3 to increase levels of the transcription factor, NRF2. This results in elevated expression of the antioxidant enzyme, glutathione peroxidase-4 (GPX4). By reducing oxidizing lipids involved in ferroptosis, cell survival and drug resistance ensue [18]. In this study, we investigated components of this mechanism for resistance via reduction of ferroptosis in drug-sensitive and drug-resistant SCLC cells, as well as the effect of the plant steroid EB on reversing resistance through the signaling pathway.

## 2. Materials and Methods

### 2.1. Cells

NCI-H69 SCLC cells (ATCC, Manassas, VA, USA), established from the pleural fluid of a male patient before treatment, were grown in suspension culture in AIM-V in 5% CO_2_ in AIM-V serum-free medium (Thermo-Fisher, Irvine, CA, USA). Cell identity was authenticated by ATCC-Promega (Madison, WI, USA). Cell cultures were mycoplasma-free, as tested by Mycofluor (Thermo-Fisher). A multi-drug-resistant cell line, VPA17, was derived from H69 cells by selection in etoposide [19] and showed resistance to etoposide (10-fold), doxorubicin (12-fold) and cisplatin (7-fold). VPA cells were tested for multi-drug resistance every 3 months and re-selected in etoposide when necessary [20]. Cells were seeded at 5 × 10^4^ cells/mL and reached logarithmic growth in 3 days, with a doubling time of 36 h.

### 2.2. Cytotoxicity

Epibrassinolide (EB); 4-hydroxynonenal (HNE); 1,3-dihyodro-1(1-4 (6-phenyl-1H-imidazo-4,5-quinoxalin-7-phenylmethyl-4-piperidinyl-benzimidazol-2-trifluoroacetate (AKTI); 1S,3R-2-(2-chloroacetyl)-2,3,4,9-tetrahydro-1-[4-(methoxycarbonyl)phenyl]-1H-pyrido [3,4-b]indole-3-carboxylic acid, methyl ester (RSL3); etoposide (ET); doxorubicin (DOX); and cisplatin (CPT) were obtained from Millipore-Sigma (St. Louis, MO, USA), dissolved in DMSO at 10 mM and stored for up to 6 months at 4 °C. Drugs were added to 0.2 mL logarithmically growing cells (0.6 × 10^4^). After 120 h incubation, cells were counted by hemocytometer, with live cells checked by Trypan Blue exclusion. Experiments were done in triplicate and repeated at least twice. The IC_50_ was defined as the concentration of additive that reduced treated cell counts by 50% compared to cells incubated in 1% DMSO. This DMSO concentration did not affect cell viability. In experiments investigating drug resistance reversal, cells were preincubated in 0.5 × IC_50_ for 48 h, after which medium was removed by centrifugation and cells were washed in fresh medium. The cells were then resuspended at 0.6 × 10^4^ cells/0.2 mL medium for evaluation of cytotoxicity in ET, DOX, or CPT as above.

### 2.3. Combination Studies

Pharmacological interactions of synergism, additivity and antagonism between added substances were investigated by the Chou–Talay method [21]. Briefly, cells were incubated in 1:1 ratio of the respective IC_50_ concentrations in combination at 0.25, 0.5, 1.0 and 2.0 × IC_50_. After 120 h incubation, cell counts were made and the combination index determined using Calcusyn software (Combosyn, Inc., Paramus, NJ, USA).

### 2.4. HNE Measurement

Cells were washed in phosphate-buffered saline (PBS) by centrifugation and resuspended in 500 uL PBS. Following sonication for 10 s, the cell suspension was further lysed by two cycles of freezing at −20 °C for 30 min followed by thawing at 25 °C. Cell extracts were stored at −20 °C for up to 2 wk and then assayed for protein by Bradford test (BioRad, Hercules, CA, USA) and HNE by ELISA (MyBioSource, San Diego, CA, USA, MBS 006597). Briefly, samples were incubated in a microtiter plate coated with the anti-HNE antibody. Cell extracts or standards were added to the plate along with HRP-conjugated HNE, initiating a competitive reaction between the HRP-HNE and HNE in the samples bound to the antibody. Following 30 min incubation, plates were washed to remove free HP and bound HRP was assayed by peroxidative activity and colorimetrically quantitated.

For experiments on the application of exogenous HNE to SCLC cells, the IC_50_ for HNE was first determined to be 20 nM for both cell lines. Following pre-incubation of cells in 10 nM HNE, cells were washed and the IC_50_ for etoposide determined as above.

### 2.5. Akt Activation and Inhibition

The activation of Akt by phosphorylation at Thr308 and Ser473 was measured by ELISA (Thermo-Fisher, Irvine CA, USA, 85-86048). Briefly, following incubation for 48 h in 1 µM EB (0.5 × IC_50_), cells were lysed and 200 µL extracts applied to microtiter plates coated with a specific antibody for either native Akt or pAkt. Following incubation for 1 h, plates were washed and an HRP-coupled secondary antibody was added for 20 min. HRP enzyme activity was determined colorimetrically. For paired samples, the colorimetric signals for total Akt and pAkt were compared to calculate the fraction of Akt as pAkt (activation).

The IC_50_ values for the Akt inhibitor (AktI) were 60 nM for H69 cells and 40 nM for VPA cells. In addition, this concentration inhibited the conversion of Akt to pAkt. The effect of incubation of cells for 96 h in 0.5 × IC_50_ AktI on GKS3β enzyme activity was determined as noted previously [16]. AktI was also tested for reversal of drug resistance, and combination analysis with EB was performed as described above.

### 2.6. NRF2 Assay

The concentration of Nrf2 was measured by cell-based ELISA (Boster, Pleasanton, CA, USA, EKC1408). Briefly, wells in a microtiter plate were incubated with 100 µL of 10 ug/mL poly-L-lysine overnight at 37 °C. Following removal of the solution, 200 µL cell suspension (4 × 10^4^ cells) was added and the plate incubated overnight at 37 °C. Microscopy showed over 90% cell attachment. Cells were treated with 1µM EB for 48 h in medium (untreated control), washed in PBS and then fixed with 8% paraformaldehyde for 20 min. Following incubation for 16 h with a primary antibody (rabbit anti-Nrf2), plates were washed in incubated for 1.5 h with an HRP-coupled secondary antibody (goat ant-rabbit) and then HRP enzyme activity was detected colorimetrically. Plates were then washed and incubated with 0.1% crystal violet solution to quantitate nucleic acids. Calculation of the ratio of absorbance at 450 nm (for Nrf2) to 595 nm (for nucleic acids) produced a relative value for Nrf2 on a cellular basis.

### 2.7. GPX4 Protein Level and Enzyme Activity

GPX4 enzyme activity in cell extracts was measured by the consumption of reduced glutathione (GSH) (Elabscience, Houston, TX, USA, E-BC-K096-M). Briefly, GSH was measured before and after the enzyme reaction by its reaction with the chromogen, 5-thio-dintrobenzoic acid, and compared with controls without an enzyme source. Protein concentration was measured as above. The specific activity of GPX4 was calculated as µmol GSH converted/5 min/mg protein.

The concentration of GPX4 protein was measured by sandwich ELISA with HRP-coupled secondary antibody detection as described above (Proteintech, Rosemont, IL, USA, KE00577). Protein was quantitated by Braford test.

The IC_50_ for RSL3, the inhibitor of GPX4, was 30 nM for both cell lines. At 15 nM (0.5 × IC_50_), RSL3 inhibited GPX4 activity in cell extracts of both cell lines (below). Following incubation of cells with 15 nM RSL3, HNE and multi-drug resistance were measured as described above. The combination index for EB and RSL3 was also estimated by the Chou–Talay method as described above.

### 2.8. Statistical Analysis

Except as noted, experiments were carried out in triplicate and repeated at least once. Data means were compared statistically by *t*-test for paired samples, with significance defines as *p* < 0.05. IC_50_ values were calculated by linear regression.

## 3. Results

Previous studies showed that the plant steroid hormone, EB, reverses multi-drug resistance in VPA-SCLC cells, but that the reversal could not be accounted for by the direct or indirect modulation of the drug transporters *pgp1* and *mrp1* [15,16]. This prompted investigations into ferroptosis and Akt signaling, which have been suggested as mechanisms for multi-drug resistance [10,18].

### 3.1. HNE Levels in Drug-Sensitive and Drug-Resistant SCLC Cells

4-hydroxynonenal (HNE) is a marker for lipid peroxidation and ferroptosis [22,23]. On a protein basis, HNE was lower in VPA drug-resistant cells than in drug-sensitive cells. Reversal of drug resistance by EB incubation led to an increase in HNE (Table 1). These effects were not observed by incubation with etoposide, a drug that is part of drug resistance. In addition, incubation of SCLC cells in exogenous HNE resulted in an increase in drug sensitivity (reversal of drug resistance) (Table 2).

Ferrostatin inhibits ferroptosis by inserting into cell membranes and reducing lipid peroxides [24]. Preincubation of both drug-sensitive H69 and drug-resistant VPA SCLC cells with ferrostatin resulted in less HNE and enhanced drug resistance (Table 3).

Ferrostatin also reversed the effects of EB noted above in Table 1. For example, the level of HNE (pg/ug) in untested VPA cells increased from 36 to 95 after treatment with EB but remained at a lower level (42 pg/ug) in VPA cells treated with EB and ferrostatin.

### 3.2. Activation and Inhibition of the AKT Pathway in SCLC Cells

Ferroptosis can be regulated through the Akt signaling pathway [22,23]. Akt is activated through phosphorylation [25]. The extent of Akt phosphorylation was greater in drug-resistant than in drug-sensitive SCLC cells (Table 4). Preincubation of cells with EB did not affect Akt phosphorylation.

The inhibitor of Akt phosphorylation, 1,3-dihyodro-1(1-4 (6-phenyl-1H-imidazo-4,5-quinoxalin-7-phenylmethyl-4-piperidinyl-benzimidazol-2-trifluoroacetate (AktI), was cytotoxic to both SCLC cell lines, with IC_50_ values of 60 nM (H69) and 40 nM (VPA). Both EB and AktI preincubations led to a reduction in GSK3β activity, an important component of Akt signaling pathways (Table 5). At 0.5 × IC_50_, AktI reversed multi-drug resistance of etoposide, doxorubicin and cisplatin, similar to EB (Table 6).

Pharmacological combination analysis by the Chou–Talay method [21] showed a Combination Index for EB and AktI of 1.22 (at ED75), possibly indicating moderate antagonism (same pathway).

### 3.3. Effects of EB and AktI on Nrf2 in H69 and VPA Cells

The transcription factor NRF2 (Nuclear factor erythroid 2-related factor 2) is a key regulator of the antioxidant response to ferroptosis [26]. On a protein basis, the concentration of NRF2 was greater in drug-resistant than in drug-sensitive SCLC cells. Preincubation of either EB or Akt1 reduced the concentration of NRF2 (Table 7).

### 3.4. GPX4 Concentration and Activity in H69 and VPA Cells

Glutathione peroxidase (GPX4) is a key antioxidant enzyme that inhibits ferroptosis by catalyzing the reduction of lipid peroxides [27]. Both the protein level and enzyme activity of GPX4 were higher in drug-resistant than drug-sensitive SCLC cells (Table 8).

### 3.5. Effects of GPX4 Inhibition on Ferroptosis and Drug Resistance

1S,3R-2-(2-chloroacetyl)-2,3,4,9-tetrahydro-1-[4-(methoxycarbonyl)phenyl]-1H-pyrido [3,4-b]indole-3-carboxylic acid, methyl ester (Ras-selective lethal-3: RSL3) binds covalently to and inhibits the enzyme activity of GPX4 [28]. The GPX4 enzyme activities of cell extracts of both H69 (drug-sensitive) and VPA (drug-resistant) SCLC were inhibited by 75% by RSL3 (1 umol GSH/min/mg protein compared to 4.2 for untreated H69 extracts; 6 umol/min/mg for treated VPA extracts compared to 23 untreated). This was accompanied by an increase in HNE, indicating ferroptosis (Table 9). In VPA but not H69 cells, the increased ferroptosis due to inhibition of GPX4 resulted in a reversal of drug resistance (Table 9).

Pharmacological combination analysis by the Chou–Talay method [21] showed a Combination Index for EB and RSL3 of 1.28 (at ED75), possibly indicating moderate antagonism (same pathway).

## 4. Discussion

**Figure 1:**
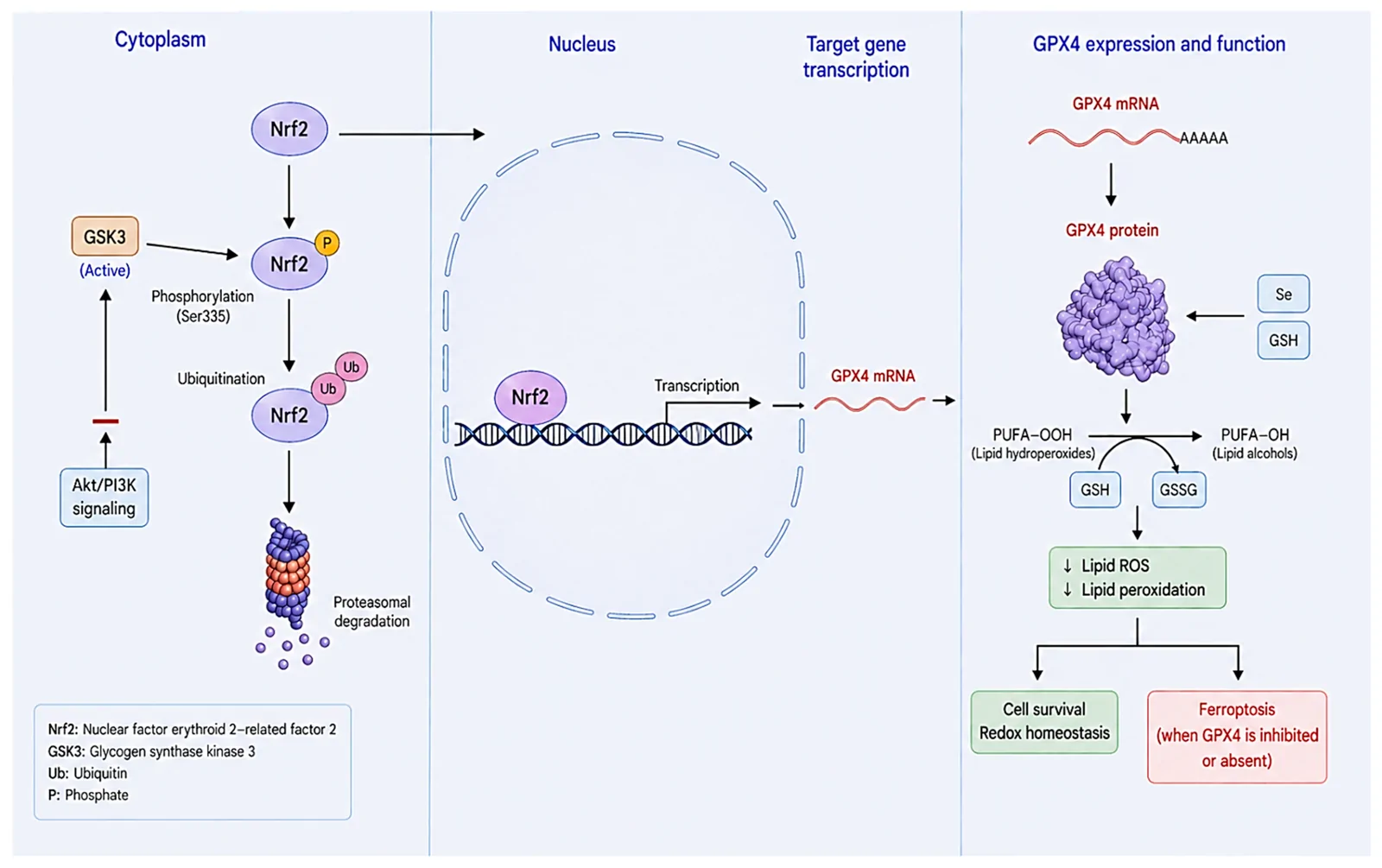
Overview of the ferroptosis pathway for drug resistance. Figure produced in part by generative AI.

Taken together, our results indicate that reduction of ferroptosis is an important mechanism for drug resistance in SCLC cells and that the plant steroid EB reverses drug resistance through modification of the Akt pathway by decreasing the activity of the antioxidant enzyme, GPX4. Figure 1 outlines the pathway involved.

Previously, we reported that SCLC cells express the transferrin receptor and accumulate Fe^2+^ [12]. The evidence for involvement of ferroptosis in SCLC drug resistance from our current studies includes the following observations: The beginning of the pathway, Akt signaling, was elevated in drug-resistant cells (Table 4). Inhibition of Akt signaling by AktI reversed drug resistance (Table 6). At the end of the pathway, accumulation of unsaturated oxidized fatty acids as evidenced by HNE was reduced in drug-resistant compared to drug-sensitive cells (Table 1). Relatedly, the application of exogenous HNE to SCLC cells made them more chemosensitive to etoposide (Table 2). These effects of HNE were inhibited by ferrostatin, again indicating that normally ferroptosis is involved in cytotoxicity and is reduced in drug resistance (Table 3).

The intermediate steps between Akt signaling and accumulation of toxic oxidized fatty acids were also examined. Akt signaling acts via autophosphorylation and effects on enzyme activity of GSK3β, and this was reduced in drug-resistant cells compared to drug-sensitive cells (Table 5). This led to an increase in the transcription factor NRF2, an increase that was Akt-dependent (Table 7). NRF2 activates the expression of the anti-oxidant enzyme, GPX4. Both protein level and enzyme activity of GPX4 were increased in drug-resistant SCLC cells (Table 8). Inhibition of GPX4 by RSL3 resulted in accumulation of more oxidized fatty acids (HNE) and increased chemosensitivity (drug resistance (Table 9)). Our data therefore confirm a pathway for drug resistance that begins with Akt activation, decreased activity of GSK3, increased NRF2 resulting in increased GPX4, and a resulting reduction in ferroptosis [18].

The ferroptosis pathway also provides an explanation for our previous observation that the plant steroid EB reverses drug resistance in SCLC cells but not through the traditional activation membrane drug efflux pumps [16]. Most notably, incubation of drug-resistant cells in EB resulted in increased ferroptosis, as measured by accumulation of HNE (Table 1). The pathway by which EB exerted this effect was the previously described inhibition by EB of GSK3β phosphorylation ([16] and Table 5). This inhibition by EB resulted in increased active GSK3β, which in turn led to a reduction in the transcription factor, NRF2 (Table 7), in turn leading to a reduction in the expression and enzyme activity of the antioxidant enzyme, GPX4 (Table 8). The accumulation of oxidized, toxic lipids resulted in increased cell death and reversal of drug resistance ([11] and Table 9). Less directly, analysis by the Chou–Talay method [21] of combinations of EB with the Akt inhibitor Akt1, and with the GPX4 inhibitor RSL3, indicated that EB possibly acts pharmacologically along the same pathway as these two inhibitors.

## 5. Conclusions

Our results indicate that ferroptosis is an important mechanism for drug resistance in SCLC, and that the plant steroid hormone, EB, reverses this resistance through the Akt-GPX4 pathway. As a natural product that is non-toxic, EB may be useful in treating drug-resistant SCLC. An important limitation of our study is that it focused on single drug-sensitive and drug-resistant cell lines and must be replicated in other SCLC cell lines. Future investigations should also include SCLC xenografts and patient-derived models.

## Figures and Tables

**Table 1:** Levels of HNE in SCLC cells and effect of EB.

HNE, pg/µg Protein			
H69 (Drug-Sensitive)		VPA (Drug-Resistant)	
Untreated	+EB	Untreated	+EB
60 ± 33 ^a^	98 ± 41	22 ± 10 ^a^	94 ± 38 ^b^

Cells were incubated with or without 1 µM EB for 96 h. Following lysis by freeze–thaw and sonication, cell extracts were evaluated for total protein and HNE by ELISA. ^a^ Significantly different (*p* < 0.05) by *t*-test comparing HNE in H69 and VPA cells. ^b^ Significantly different (*p* < 0.05) by *t*-test comparing untreated and treated cells.

**Table 2:** Effect of exogenous HNE on cytotoxicity of etoposide on SCLC cells.

IC_50_ for Etoposide, µM			
H69 (Drug-Sensitive)		VPA (Drug-Resistant)	
Untreated	+HNE	Untreated	+HNE
0.22 ± 0.08 ^a^	0.05 ± 0.03 ^b^	7.5 ± 3.6 ^a^	1.0 ± 0.4 ^a,b^

Cells were pre-incubated with or without 0.5 × IC_50_ of HNE for 48 h. Following removal of HNE by washing, cytotoxicity of etoposide was evaluated after 5 days. ^a^ Significantly different (*p* < 0.05) by *t*-test comparing IC_50_ in H69 and VPA cells. ^b^ Significantly different (*p* < 0.05) by *t*-test comparing untreated and treated cells.

**Table 3:** Effect of ferrostatin on HNE and cytotoxicity of etoposide on SCLC cells.

	H69 (Drug-Sensitive)		VPA (Drug-Resistant)	
	Untreated	+Ferrostatin	Untreated	+Ferrostatin
HNE, pg/µg	54 ± 16 ^a^	21 ± 8 ^b^	19 ± 14 ^a^	6 ± 4 ^b^
IC_50_ etop, µM	0.6 ± 0.2 ^a^	4.8 ± 2.2 ^b^	5.5 ± 1.8 ^a^	18 ± 5.6 ^b^

Cells were pre-incubated with or without 0.5 × IC_50_ of ferrostatin for 48 h. Following removal of ferrostatin by washing, HNE was measured by ELISA and cytotoxicity of etoposide was evaluated after 5 days. ^a^ Significantly different (*p* < 0.05) by *t*-test comparing H69 and VPA cells. ^b^ Significantly different (*p* < 0.05) by *t*-test comparing untreated and treated cells.

**Table 4:** Activation of Akt in SCLC cells.

ELISA OD_450nm_		
	H69 (Drug-Sensitive)	VPA (Drug-Resistant)
Akt total	0.21 ± 0.10	0.30 ± 017
pAkt	0.08 ± 0.05	0.27 ± 0.20
% pAkt	38	90 ^a^

Cell extracts (100 µg protein) were assayed initially for total Akt by ELISA, followed by a separate ELISA for pAkt. ^a^ Significantly different (*p* < 0.05) by *t*-test comparing H69 and VPA cells.

**Table 5:** Effect of inhibition of Akt phosphorylation (AktI) and EB on activity of GSK3β in SCLC cells.

Luminescence × 10^4^ (% Activity)		
	H69 (Drug-Sensitive)	VPA (Drug-Resistant)
Untreated	3.5 ± 2.1 (100%)	2.5 ± 1.1 (100%)
EB	6.4 ± 1.8 ^a^ (33%)	5.6 ± 2.2 ^a^ (44%)
AktI	7.1 ± 3.3 ^a^ (30%)	6.0 ± 2.8 ^a^ (42%)

Cells were pre-incubated with or without 0.5 × IC_50_ of EB or AktI for 48 h. Following removal of the treatment by washing, GSK3β activity in cell extracts was measured by luminescence [16]. ^a^ Significantly different (*p* < 0.05) by *t*-test comparing untreated and treated cells.

**Table 6:** Effect of inhibition of Akt phosphorylation (AktI) and EB on multi-drug resistance in SCLC cells.

IC_50_,µM		
	H69 (Drug-Sensitive)	VPA (Drug-Resistant)
Etoposide	0.4 ± 0.2	6.0 ± 2.4
Etop + EB	0.4 ± 0.3	1.5 ± 0.8 ^a^
Etop + AktI	0.7 ± 0.6	2.3 ± 0.8 ^a^
Doxorubicin	0.05 ± 0.03	0.37 ± 0.15
Dox + EB	0.05 ± 0.04	0.11 ± 0.07 ^a^
Dox + AktI	0.02 ± 0.01	0.05 ± 0.02 ^a^
Cisplatin	1.2 ± 0.4	9.5 ± 3.5
Cispt + EB	1.5 ± 0.7	2.0 ± 0.9 ^a^
Cispt + AktI	2.0 ± 0.9	2.2 ± 1.0 ^a^

Cells were pre-incubated with or without 0.5 × IC_50_ of EB or AktI for 48 h. Following removal of the treatment by washing, cytotoxicity of indicated drugs was determined after 5 days of incubation. ^a^ Significantly different (*p* < 0.05) by *t*-test comparing drug IC_50_ for preincubated cells and untreated cells.

**Table 7:** Effect of inhibition of Akt phosphorylation (AktI) and EB on concentration of NRF2 in SCLC cells.

NRF2:Protein Ratio OD_450_/OD_595_		
	H69 (Drug-Sensitive)	VPA (Drug-Resistant)
Untreated	0.67 ± 0.42	1.45 ± 0.36 ^a^
EB	0.39 ± 0.11 ^b^	0.57 ± 0.22 ^b^
AktI	0.34 ± 0.28 ^b^	0.48 ± 0.20 ^b^

Cells were pre-incubated with or without 0.5 × IC_50_ of EB or AktI for 48 h. Following removal of the treatment by washing, NRF2 and total protein were measured by ELISA. ^a^ Significantly different (*p* < 0.05) by *t*-test comparing H69 and VPA cells. ^b^ Significantly different (*p* < 0.05) by *t*-test comparing untreated and treated cells.

**Table 8:** Effect of EB on GPX4 concentration and enzyme activity in SCLC cells.

	H69 (Drug-Sensitive)	VPA (Drug-Resistant)
GPX4 concentration		
pg/mg protein:		
Untreated	10.1 ± 4.6	21.2 ± 7.5 ^a^
+EB	2.5 ± 1.0	6.8 ± 4.1 ^a^
GPX4 enzyme act.		
µmol/mgprot/5 min:		
Untreated	32 ± 14	96 ± 22 ^a^
+EB	15 ± 9	28 ± 16 ^a^

Cells were pre-incubated with or without 0.5 × IC_50_ of EB 48 h. Following removal of the treatment by washing, GPX4 protein level was measured by ELISA. GPX4 enzyme activity was measured colorimetrically. ^a^ Significantly different (*p* < 0.05) by *t*-test comparing H69 and VPA cells.

**Table 9:** Effect of RSL3 inhibition of GPX3 on HNE and cytotoxicity of etoposide on SCLC cells.

	H69 (Drug-Sensitive)		VPA (Drug-Resistant)	
	Untreated	+RSL3	Untreated	+RSL3
HNE, pg/µg	95 ± 44 ^a^	245 ± 101 ^b^	40 ± 19 ^a^	175 ± 90 ^b^
IC_50_ etop, µM	0.4 ± 0.2 ^a^	0.5 ± 0.3 ^b^	6.5 ± 1.8 ^a^	0.8 ± 0.3 ^b^

Cells were pre-incubated with or without 0.5 × IC_50_ of RSL3 for 48 h. Following removal of RSl3 by washing, HNE was measured by ELISA and cytotoxicity of etoposide was evaluated after 5 days. ^a^ Significantly different (*p* < 0.05) by *t*-test comparing H69 and VPA cells. ^b^ Significantly different (*p* < 0.05) by *t*-test comparing untreated and treated cells.

## Data Availability

The original contributions presented in this study are included in the article. Further information can be obtained from the authors.
